# Raman Plus X: Biomedical Applications of Multimodal Raman Spectroscopy

**DOI:** 10.3390/s17071592

**Published:** 2017-07-07

**Authors:** Nandan K. Das, Yichuan Dai, Peng Liu, Chuanzhen Hu, Lieshu Tong, Xiaoya Chen, Zachary J. Smith

**Affiliations:** Department of Precision Machinery and Precision Instrumentation, University of Science and Technology of China, 96 Jinzhai Road, Hefei 230027, China; nandan@mail.ustc.edu.cn (N.K.D.); daiyc@mail.ustc.edu.cn (Y.D.); lpeng01@ustc.edu.cn (P.L.); hczstu@mail.ustc.edu.cn (C.H.); tongls@mail.ustc.edu.cn (L.T.); afra@mail.ustc.edu.cn (X.C.)

**Keywords:** Raman spectroscopy, multimodal, light scattering

## Abstract

Raman spectroscopy is a label-free method of obtaining detailed chemical information about samples. Its compatibility with living tissue makes it an attractive choice for biomedical analysis, yet its translation from a research tool to a clinical tool has been slow, hampered by fundamental Raman scattering issues such as long integration times and limited penetration depth. In this review we detail the how combining Raman spectroscopy with other techniques yields multimodal instruments that can help to surmount the translational barriers faced by Raman alone. We review Raman combined with several optical and non-optical methods, including fluorescence, elastic scattering, OCT, phase imaging, and mass spectrometry. In each section we highlight the power of each combination along with a brief history and presentation of representative results. Finally, we conclude with a perspective detailing both benefits and challenges for multimodal Raman measurements, and give thoughts on future directions in the field.

## 1. Introduction

Raman spectroscopy is an extraordinarily powerful tool for characterizing the chemical content of samples, ranging from pharmaceuticals [1,2,3], industrial process components [4,5], ancient art and pigments [6,7], and, of course, a wide array of biological and biomedical samples (as detailed by the research articles contained elsewhere in this special issue) [8,9,10]. This wide range of applications, combined with the unique advantages of Raman spectroscopy as a label-free and non-destructive technique, helps to explain the rapid and continued growth of the Raman spectroscopic community over the past few decades. Due to its non-invasiveness, there has been persistent interest in using Raman spectroscopy to diagnose disease at the tissue level without the need for a physical biopsy of the sample [11]. Its label-free nature also makes Raman spectroscopy a natural choice for examining biochemical changes within cells due to drug interactions, immunogenic activation, and other dynamic stimuli [12]. Due to its extreme sensitivity to minor chemical changes within a sample, Raman spectroscopy, when used alone, often outperforms other spectroscopic methods in its ability to diagnose disease states or track the biochemical content of a cell over time [13].

However, no technique is an ideal solution for all problems, and Raman spectroscopy also has its own well-known limitations. The classic drawback of Raman spectroscopy is its lack of signal strength. Due to the very small scattering cross section for most molecules, Raman signals tend to be several orders of magnitude weaker than fluorescence or elastic scattering signals. This potentially limits the addressable application space for Raman spectroscopy. For example, acquisition times of limited fields of view on the order of several minutes to hours would not be appropriate for determining tumor margins intraoperatively. Or monitoring cellular uptake of a small molecule agonist with fast kinetics would not be possible with a Raman system that can only achieve a 2-min frame refresh rate. Another drawback is that Raman spectroscopy, when applied to most biological samples such as cells and tissues, is only able to obtain information about large classes of biomolecules (such as lipids, nucleic acids, proteins) without necessarily providing the molecular specificity most biologists are used to achieving using antibody-conjugated fluorophore systems. Finally, while Raman scattering is highly sensitive to chemical changes, due to the molecular origin of the scattering signature Raman spectroscopy is at best weakly sensitive to the structure of a sample at length scales beyond the molecular scale. Thus, to obtain morphological information (or indeed any information beyond chemical content), other modalities must be employed.

To overcome these drawbacks, or simply to provide additional information orthogonal to that provided by standard Raman measurements, there has been a long effort of incorporating Raman spectroscopy into multimodal systems, where multiple measurements are made on the same sample by different methods. Integrating Raman with other modalities requires careful consideration, for example different modalities may probe different regions of the sample, or require special sample preparation above and beyond those considerations of Raman spectroscopy alone. Further, careful consideration of the relative unique advantages and disadvantages each mode brings to a particular problem is necessary to ensure that the ultimate multimodal system is a logical combination rather than an “instrument to nowhere”. However, when this is done correctly, the results can significantly enhance the performance and applicability of the combined multimodal measurement over what either modality could have provided alone. In this review, we discuss the combination of Raman scattering with several other optical modalities, including fluorescence, elastic scattering, and OCT. We also briefly discuss the integration of Raman with non-optical modalities such as mass spectrometry. Finally, in our concluding section, we offer several thoughts on the future opportunities and challenges to the combination of Raman with other modalities for biological applications.

## 2. Raman Plus Fluorescence

Raman and fluorescence at first glance may seem to be unlikely bedfellows. Raman experts are intimately familiar with the great lengths to which most experiments must go to avoid fluorescence contamination of the Raman signal [14]. Indeed expensive and complex instruments are continually being designed to allow researchers the ability to record Raman spectra of highly fluorescent samples, either through ultrafast gating [15,16,17] or wavelength-modulated excitation [18,19,20]. Further, both Raman and fluorescence report on the chemical content of the sample. Therefore, one might conclude that these should be adversarial rather than cooperative methods. In many ways this has been the case: in the field of spectroscopy-based disease diagnosis, there are several publications exploring the relative utility of tissue fluorescence versus tissue Raman spectroscopy for cancer diagnosis, among other topics [13,21,22]. It was not until 2005 that Zeng and co-workers demonstrated that combining autofluorescence and Raman spectroscopy results in more accurate diagnostics than using either modality alone [23]. Other early work on combined Raman and fluorescence imaging systems tend to use fluorescence as a “confirmatory” step to demonstrate that the conclusions reached via Raman-based imaging are accurate [24]. However, for many problems, such as those described below, Raman and fluorescence can be a powerful combination. In 2009, Šćepanović et al. developed a tri-modal, fiber-based, probe system for disease diagnostics. In this system, Raman is combined with fluorescence, and also with diffuse reflectance (representing both scattering and absorption features in diffuse tissues) [25]. This system was then used to diagnose vulnerable and thrombotic plaques from ex vivo human arteries [26]. The overall sensitivity of diagnosis with the tri-modal system was substantially higher than with Raman alone (96% versus 79%), conclusively demonstrating the benefits of the multimodal measurements. 

**Figure 1:**
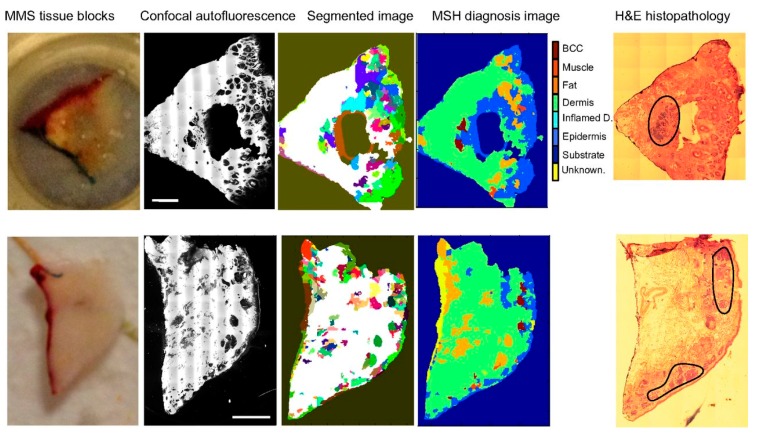
Multimodal Spectral Histology (MSH) diagnosis of BCC in unsectioned tissue blocks as received from surgery. H&E histopathology images for adjacent sections are included for comparison. (Scale bars: 2 mm.) Figure reproduced from Kong et al., [28].

When comparing Raman and fluorescence, fluorescence has the advantages of strong signals and molecular specificity, but in part due to this specificity, fluorescence measurements can only probe a narrow class of biomolecules. This generally limits the ability of tissue auto-fluorescence to perform an automated diagnosis. Raman, by contrast, generally has exquisite ability to diagnose tissues, but slow measurement speeds limit its ability to rapidly image a macroscopic field of view. Thus, fluorescence may be able to provide a rapid method to isolate defined regions of interest for detailed Raman analysis. This approach was pioneered as far back as 2003 by Otto and colleagues for cellular analysis [27]. An excellent case study for the power of this approach is a recent publication by Kong et al., where Raman imaging was combined with autofluorescence imaging of excised cancer tissue during Mohs surgery [28], as shown in Figure 1. In the excised cancer tissue it is critical to obtain a rapid segmentation of the image into cancerous and noncancerous regions, to ensure that the margins of the excised tissue are free from tumor infiltration (otherwise the surgeon must go back and make a second cut). Autofluorescence has been long explored as a method to separate cancerous and noncancerous tissues. It has the significant advantage of being label free, fast, and with strong signals. However, it has weak specificity for cancer. Raman, on the other hand, has high sensitivity and specificity for cancer versus normal tissues, yet its slow speed make it impractical for use in a tissue-conserving Mohs surgery context. Kong and co-authors make use of the advantages of both autofluorescence and Raman imaging, while mitigating the drawbacks of both. In their methodology, an autofluorescence image is used to segment images of thin basal cell carcinoma sections, eliminating wide swaths of the image from needing to be measured by Raman spectroscopy and grouping the remaining tissue into larger segments. Each segment is measured at several points and its diagnosis (healthy versus tumor) is determined. In this way, the entire surface of the excised tissue can be quickly classified, speeding up the measurement of the tissue by more than 100 times, while not degrading the diagnostic accuracy. This work has recently been expanded to other cancer types and is likely to become a powerful method for multimodal diagnosis of other diseases as well [29,30,31,32].

In another recent work by Carney et al., Raman spectroscopy was used in combination with fluorescence to identify the biochemical differences of distinct subpopulations of individual extracellular vesicles [33]. Extracellular vesicles are small nanosized constructs released by cells as part of their communication with their extracellular environment. These vesicles have distinct membrane constructions, which are highly enriched in targeting proteins, and their cargo (primarily active RNA and proteins) is actively packaged by the cell, suggesting that vesicle contents are likely to be highly heterogeneous depending on the intended function of the vesicle (cell communication, immune regulation, etc.). In a previous report, Smith et al. showed that Raman spectroscopy was able to record Raman spectra from single vesicles, and that the spectra viewed on the single vesicle level revealed a surprising amount of heterogeneity, particularly in the membrane construction [34]. However, because Raman spectroscopy lacks the molecular specificity to identify surface protein markers on the exosomes, the functional significance of this heterogeneity remained to be investigated. Carney et al., by labeling the CD9+ subpopulation of extracellular vesicles using fluorescent-conjugated antibodies, were able to separately identify the Raman spectra of the “average” extracellular vesicle released by several cell lines, and contrast that with the Raman spectra of the CD9+ vesicles, clearly showing a marked difference in the total biochemical content as well as chemical makeup. Thus, by combining the broad, label-free biochemical information provided by Raman spectroscopy, and the molecular specificity of fluorescence, the multimodal measurements system marks a significant step forward in unraveling the origin and function of diverse vesicle populations.

The combination of Raman and fluorescence has also been explored in Surface Enhanced Raman Spectroscopy (SERS)-based biosensors. Kang et al. reported an innovative combination of SERS and fluorescence for visualization of pH-dependent drug delivery [35]. By conjugating doxorubicin to a gold nanoparticle (AuNP) via a pH dependent linkage, the authors were able to quench doxorubicin’s native fluorescence. Thus, in neutral pH, the doxorubicin-AuNP complex yielded strong and specific Raman signals. However, when the complex entered a low-pH environment (such as an intracellular lysosome), the doxorubicin was freed from the AuNP and its fluorescence was clearly visible. Thus, by tracking the Raman versus fluorescence signal from the doxorubicin, the intracellular drug distribution and local pH were simultaneously recorded. With this hybrid system, the temporal dynamics of the drug uptake, lysosomal translocation, and subsequent release into the cell cytoplasm was able to be uniquely and completely determined. A related technique was developed recently by Jeong et al., where a fluorescent-SERS hybrid nanoparticle reporter was developed for endoscopic applications [36]. By designing a nanoparticle that emits spectrally distinct SERS and fluorescent signals excited by a single excitation source, the fluorescence emission can be used to rapidly localize individual nanoparticles targeted to HER2 or EGFR. Subsequent SERS analysis provides the ability to distinguish individual tissue types.

## 3. Raman Plus Elastic Light Scattering

In contrast to Raman scattering, where energy is transferred between the photon and the molecule, in elastic scattering there is no energy transfer, and the redirection of light happens primarily due to index of refraction differences that can span multiple length scales. Because elastic scattering does not probe the energy structure of the scattering material, it has very little sensitivity to chemical content of the sample (other than a weak dependence on the refractive index). Rather, it reports on the morphological structure of the sample. Thus, elastic scattering can be used, for example, to determine the sizes of cells in suspension [37], the size of organelles within a cell [38,39], the stochastic length scale of the molecular scale disorder [40,41,42], the organization of vessels within healthy and cancerous tissues [43], among myriad other uses outside of biology. Elastic scattering is essentially an interferometric technique, and can provide information on scatterers much smaller than the diffraction limit, and with much higher precision than traditional imaging can provide. For example, elastic scattering measurements performed with extremely low-cost sources and detectors from a consumer-grade cell phone have been shown to have ~15 nanometer precision in sizing polystyrene size standards and suspensions of cells and lipid droplets [44].

Here we focus primarily on two combinations of Raman and elastic scattering. In the first, dubbed Spatial Offset Raman Spectroscopy (SORS) [45], elastic scattering plays an adjunct role. Light is launched into a diffusing medium and Raman light is collected by a spatially offset detector. Due to scattering within the medium, the offset detector tends to sample increasingly deep tissue layers with increasing distance from the source. Scattering prolongs the interaction length between the laser and sample, increasing Raman signals despite significant losses due to diffusion. In the second, Raman and elastic scattering measurements are combined to simultaneously characterize the morphology and chemical content of a sample without labels and with subcellular resolution [46]. Here researchers exploit the unique information content provided by Raman and elastic scattering produced by the same laser excitation.

### 3.1. Spatial Offset Raman Spectroscopy

Measurements of subsurface structures embedded within scattering media are of critical importance in biomedical optics. Cancers, for example, often arise at the dermal/epidermal junction in tissue, residing several hundred microns below the surface. Additionally, noninvasive measurements of the matrix composition of subcutaneous bone, measurements of blood oxygenation in subsurface vasculature, and other similar medical questions require delivering and collecting light from deep structures covered by highly scattering tissues. Furthermore, many medicines are composed of a layered structure, where pharmacologically active compounds are embedded in a highly scattering supporting matrix, and then coated with other chemicals to delay or otherwise enhance the drug’s pharmacokinetics. The typical approach to recover Raman signals from deep-lying structures is to use a confocal system, where only light that travels ballistic paths to and from the subsurface sample plane are passed through the optical system with high efficiency [47]. However, confocal has a limited penetration depth of between 100 and 200 microns. More recent advances in phase conjugation allow this depth to be extended but at the cost of significant complexity, and furthermore only improve the penetration depth to 1 to 2 mm [48,49].

In the case of diffuse photon transport, the “culprit” behind the diffusion is light being multiply scattered. Thus, elastic scattering in some sense is playing a detrimental role in diffuse tissues. However, in the early 2000s, Matousek at Rutherford Appleton Labs realized that the case is not so clear cut [45]. While diffusion reduces the ability for photons to travel ballistic paths, the “snake” paths that photons do tend to take in diffuse tissue actually have the property of increasing the potential interaction pathlength between the incident laser photons and the material [50]. This has been theoretically studied by Hokr and Yakovlev [51] who showed that the enhancement provided by elastic scattering is robust over a wide range of scattering media. However, their conclusion is that SORS is best-suited to mildly scattering media (mean scattering length >0.1 mm), while highly scattering media may be best suited using a time-gated or other more complex approach [15,52]. Maher and Berger also demonstrated that for each application, where sample turbidity and Raman signal strength are varying, there is an ideal offset between source and detector to provide the maximum signal-to-noise ratio [53]. Thus, when a system is appropriately constructed, a measurement can be made that takes advantage of the diffusion in the system to substantially enhance the Raman signal from underlying layers, while minimizing interference from overlying layers. Furthermore, when these overlying layers are highly fluorescent, this method allows for effective suppression of fluorescence contamination of the Raman spectra of the lower layer [54,55]. This technique, which borrows heavily from standard diffuse optical spectroscopy instrumentation [56], is dubbed Spatially Offset Raman Spectroscopy.

One of the earliest demonstrations of SORS was in pharmaceutical applications, where the layered, scattering structure of pills and packaging made it ideal for SORS. In two papers, Ricci et al. and Eliasson and Matousek demonstrated that SORS can authenticate medications, such as fake and genuine anti-malarial tablets, directly through packaging [57,58], allowing fully nondestructive testing of medicines without even damaging the packaging. Olds et al. also demonstrated SORS for the detection of concealed drugs like dissolved acetaminophen in methanol inside a plastic bottle and postal packages or envelopes concealing white powders [59]. 

Another common application of SORS is in the study of bone quality. Conventional Raman spectroscopy has been widely used to analyze bone properties, such as bone age, biomechanical status and composition variation related to bone diseases [60,61,62,63]. SORS, as an effective tool for non-invasive transcutaneous characterization, is able to collect Raman spectra of bones through several millimeters of soft tissue. Schulmerich et al. [64] first achieved this in 2006 in both chicken tibia and a human elbow. The team used a uniformly illuminated array of collection fibers to increase the signal and multivariate data reduction to resolve the Raman spectra. The first comparison of transcutaneous and exposed bone measurements in vivo on humans was conducted by Esmonde-White et al. [65], with the results demonstrating the feasibility of SORS for transcutaneous bone characterization. Buckley et al. utilized SORS in diagnosis of osteogenesis imperfecta [66] and showed the potential of sensitive prediction of fragility fractures [67]. Maher, Berger, and co-workers subsequently demonstrated that SORS can provide a non-invasive method to characterize bone fragility in a mouse model of osteoporosis [68,69,70], as well as demonstrating that SORS can further predict glucocorticoid-induced changes bones quality [71,72,73,74]. While SORS is most commonly used for transcutaneous measurements, recent results demonstrate that SORS measurements on exposed bone (for example during surgery) can provide enhanced sensitivity to subcortical bone and marrow [75].

Finally, SORS has also been used to probe for cancers residing deep within tissue, such as in breast cancer. Currently, mammography is a commonly used method of breast cancer diagnosis, but it is not able to diagnose whether the lesion is benign or malignant. Discriminating microcalcifications into Type I or Type II is important, since Type I always indicates a benign lesion, whereas Type II indicates both benign and malignant lesions [76,77]. As the work in bone discussed above demonstrates, SORS has excellent sensitivity to calcium compounds lying deep within tissue. Thus, it is a promising tool to differentiate Type I (consisting of calcium phosphates) and Type II (consisting of calcium hydroxyapatite) based on the Raman spectra. Stone et al. [78] utilized SORS in calcification probing and first demonstrate its potential for in vivo breast analysis, with up to two orders of magnitude deeper penetration than conventional Raman spectroscopy. Later, Keller et al. demonstrated conclusively that SORS can be used to distinguish between multiple layers of soft tissues [79], and further designed a SORS probe for intraoperative breast cancer margin detection. For the SORS probe, Monte Carlo simulations were used to help characterize the relation between the offset detection distance of SORS, detectable tumor size and its depth [80]. Keller further designed a multiple source-detector probe that was tested on frozen–thawed breast tissue samples, achieving a margin detection result of 95% sensitivity and 100% specificity [81]. 

Other methodological improvements to SORS are continually being reported in the literature. For example, Liao et al. recently demonstrated that utilizing a DMD in place of delivery or collection fibers, arbitrary illumination and collection configurations can be easily and flexibly applied, substantially simplifying SORS setups [82]. Stone et al. also demonstrated a related technique to SORS called “enhanced transmission Raman spectroscopy”, where a dielectric bandpass filter is placed on top of tissue, with the effect of reflecting backscattered light at the laser wavelength back into tissue. In so doing, the filter gives elastically scattered light a dramatically increased effective path length within tissue. Collection optics on the other side of the tissue capture Raman photons, with signal enhancements of >200, corresponding to a 15 times improvement in SNR [83]. Further, Stone et al. have combined surface-enhanced Raman scattering (SERS) with SORS in order to demonstrate that Raman signals of deeply embedded SERS reporters could be recovered (and spatially separated) in tissue up to 4 cm thick [84].

### 3.2. Integrated Raman and Angular Scattering

Elastic scattering has a long and rich history of providing nanoscale morphometric measurements using light as a convenient, noncontact probing method [37,85,86,87]. The dominant form of elastic light scattering instruments perform bulk measurements on liquid suspensions of particles. However, in recent years there has been an increasing interest in developing instrumentation that incorporates light scattering into microscopic imaging and spectroscopy [85]. Incorporating Raman spectroscopy into these elastic scattering systems is a logical advancement. Raman spectroscopy requires a highly monochromatic source, and, due to the fact that Raman cross sections are extremely weak, the laser must be quite powerful. Therefore, abundant elastic scattering is already being generated in a typical Raman scattering measurement, but is typically removed from the measurement path and disregarded. Thus, by merely recapturing a portion of this discarded elastic scattered light, a new system can be constructed which combines the chemical specificity of Raman scattering with the detailed morphological information provided by elastic scattering.

**Figure 2:**
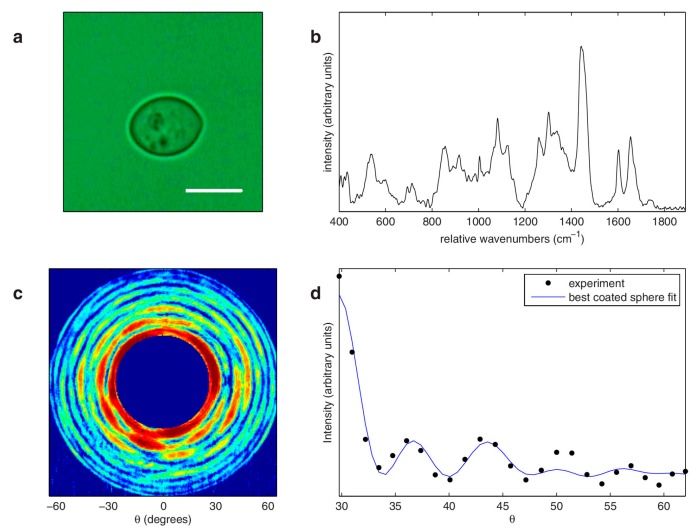
IRAM measurements of Saccharomyces cerevisiae. (**a**) Bright field image of the measured yeast cell. Scale bar indicates 5  μm. (**b**) Raman spectrum from the yeast cell shown in (**a**). (**c**) Elastic scattering pattern from the yeast cell (color scale runs from blue (low) to red (high)). (**d**) Azimuthally averaged data from (**c**) (black dots) and associated best fit using a coated sphere model (solid blue line), with extracted core and coat diameters of 6.06 and 6.22  μm, respectively. Reprinted from Smith et al., [94], with the permission from AIP Publishing.

The first combination of Raman with elastic scattering for simultaneous size and refractive index information are several contemporaneous publications by Kiefer et al. from the University of Würzburg and Kaiser et al. from Ruhr-University Bochum [88,89,90]. In these three papers, by optically levitating a single droplet, simultaneous Raman and elastic scattering information can record the chemical composition, size, and refractive index of the droplet. Interestingly, size-dependent resonances in the Raman scattering spectrum are clearly observed and can themselves provide key feedback about the evaporation and other dynamic processes within the droplet. Kiefer and colleagues further demonstrated that this experimental design could be used to monitor complex polymerization reactions occurring within single droplets [90]. In these papers, the elastic light scattering was monitored at a single angle, as is common in flow systems. Veselovskii et al. later explored the theoretical underpinnings of the size-dependent resonances observed earlier [91,92]. However, measuring the light scattering intensity at multiple angles can provide increased information about the morphology of the sample, particularly when this morphology has complex refractive index variations such as in a biological cell [38,39,87]. In 2008, Smith and Berger at the University of Rochester constructed an Integrated Raman- and Angular-scattering Microscope (IRAM), which modifies a typical Raman microspectroscopy setup to include an elastic scattering arm that measures a wide range of scattering angles in a single shot [46]. The elastic scattering arm images the Fourier plane of the microscope objective, such that the angular distribution of forward-scattered light at the laser wavelength is imaged onto a CCD. In the Fourier plane, x and y positions on the camera correspond to the θ and φ scattering angles in the sample plane. Because this system uses a tightly focused laser beam, Generalized Lorenz-Mie Theory (GLMT) must be used to account for the focused beam. The IRAM system is able to obtain nanometer sensitive information about subcellular organelles as well as the complimentary chemical information provided by Raman measurements from the same sample. The IRAM multimodal system has been shown to accurately extract information about multi-component multi-particle suspensions (including bacteria), can easily distinguish different immune cell types [93], and can provide size information about morphologically complex cells such as determining the thickness of a yeast cell wall [94], as shown in Figure 2. Later work has shown that Raman and elastic scattering can jointly describe the morphological and chemical changes that occur during immune cell activation, and can do so in a label-free manner [12]. Because both Raman and elastic scattering are “intrinsic” signals, their measurement does not impact cell dynamics, making it an ideal system for longitudinal experiments.

In 2011, Shipp et al. modified the IRAM system to allow just such long-term measurements of repeatedly measured cells [95]. This was then applied to observe cells undergoing photodynamic therapy. EMT6 cells treated with ALA (a photosensitizing agent) were exposed to 200 s of PDT therapeutic illumination. Following the treated and untreated cells over several hours, elastic scattering and Raman scattering showed significant evolution in PDT treated cells over time. The various IRAM parameters show initial decreases in large scatterer size and protein concentration. Shortly after these decreases begin, the concentration of nucleic acid also begins to decrease. Lipid concentrations begin to decrease about 3 to 6 hours after treatment [96]. Concurrently observing both subtle chemical and morphological alterations to cells after treatment could be later applied in chemotherapeutic regime optimization or to other dynamic processes such as cell division.

## 4. Raman Plus OCT

Optical coherence tomography (OCT) is an established medical imaging method which can serve as a noninvasive imaging technique with high spatial resolution and better depth penetration in biological tissue [97,98,99,100]. The working principle of the OCT technique is based on low coherence interferometry. Reflection signals from a particular internal layer of the tissue sample interferes with a reference signal whose optical path difference with sample is within the coherence length of the source. OCT has a fundamental decoupling between lateral and axial resolution, with axial resolution being coherence-limited (and thus potentially much better than optically limited resolution). OCT systems typically use near infrared light, allowing them to capture structural information from deep within tissue. Depending upon the bandwidth of the source spectra, optical coherence tomography can achieve sub-micron resolution. For example, backscattering spectral interferometry has been developed based on the principle of OCT, which can achieve resolutions of ~0.7 µm [40]. In medical diagnostics, a real-time OCT system has been developed successfully to detect ovarian cancer based on structural information of biological tissue [101,102]. However, OCT fundamentally reports on tissue structure, since its signal primarily comes from refractive index boundaries within layered tissues. Thus, complementing OCT with Raman spectroscopy, which can provide chemical interpretation, can help improve OCT results within clinical practice.

Significant interest around combined Raman-OCT systems has arisen recently to study biochemical and morphological information simultaneously for both biological and clinical applications. A joint Raman-OCT system was first demonstrated by Ko et al. [103] who sequentially recorded OCT images and Raman signals from ex vivo dental tissue to understand morphological and biochemical information simultaneously [103]. This initial work has given way to work by Patil et al. who have developed a combined platform to detect Raman and OCT information simultaneously [104]. They also developed a combined frequency domain OCT and Raman system with common probe to monitor human skin [105], showing that a hand-held Raman-OCT probe can characterize cancerous skin lesion in clinical setting [106]. 

One example of Raman plus OCT is in cancer margin detection. Incomplete resection is the main cause of the local recurrence during the carcinoma removing surgery [107,108]. Therefore, accurate detection of the boundary between cancerous and healthy regions is required for reducing the local recurrence to avoid repeated surgeries. Raman spectroscopy has been widely used in the examination of cancer pathology with high classification accuracy [109,110,111]. However, because of the weak nature of Raman scattering, it is impossible to implement a rapid spectral imaging over a large spatial area and generally point measurements is implemented instead. Sequential or simultaneous detection of Raman and OCT signals can enhance structural and molecular specificity of any biological tissue. Initial research shows that integrated Raman and OCT systems are a promising tool for detecting and classifying skin cancer successfully [106]. Work by Patil demonstrated that the combination of Raman and OCT can improve the detection accuracy of ex vivo breast cancer [104]. Ashok et al. have reported a multimodal analysis applied to colonic adenocarcinoma [112], where the structural properties were quantified from the OCT images and biomolecular properties were quantified using Raman spectroscopy. The results of the combined method were evaluated by a binary support vector machine (SVM) classification with the combined Raman/OCT classification having improved detection accuracy compared with Raman alone.

**Figure 3:**
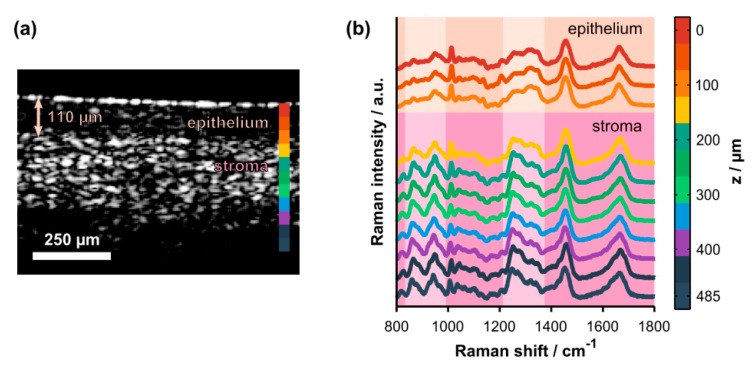
(**a**) Representative cross-sectional OCT image of intact, ex vivo porcine vaginal tissue. The arrow highlights the thickness of the superficial epithelium. The colorbar shows the axial locations where Raman spectra were acquired. (**b**) Co-localized, depth-resolved Raman spectra acquired from the same tissue sample (offset for clarity). Spectroscopic differences due to natural biochemical variation (e.g., collagen content) in the epithelium versus the stroma are highlighted by the pale-colored columns. The highlighted spectral regions include hydroxyproline and proline bands (800–1000 cm^−1^) and the amide III protein band (1200–1400 cm^−1^). Figure reproduced with permission from Maher et al., [115], Copyright 2015, Optical Society of America.

Although, many researchers have developed Raman-OCT instrumentation for a variety of medical diagnostics, one disadvantage of the above studies is that Raman spectra and OCT imaging come from different layers of the tissue having different biomolecular information. Raman information in these cases has a diffuse origin and provides average behavior only, while OCT gives morphological information of each layers separately [113]. Therefore, measuring depth-wise Raman signal may provide layered detail about biochemical information that more closely corresponds to the morphological information detected via OCT. Along these lines, Evans et al. developed a Raman-OCT system for obtaining simultaneous morphological and biochemical information of human retina [114]. They used confocal microspectroscopy to acquire depth-wise Raman spectra, with corresponding OCT structural information with penetration depth up to 2 mm. As demonstrated schematically in Figure 3, Maher et al. utilized combined Raman and OCT measurement to evaluate the penetration profile and temporal kinetics of tenofovir, a drug used to prevent HIV transmission [115]. By acquiring depth-resolved confocal Raman spectra, the concentration of the drug in depth and time could be accurately acquired with a 0.03% root-mean-squared error. Further, by correlating depths with morphological information, drug distribution in the epithelium and stroma could be separately characterized. Preliminary results also demonstrate differences in diffusion of the drug in vaginal vs. rectal tissues that can be explained by different epithelial thicknesses, highlighting the strengthened conclusions that can be drawn by acquiring simultaneous morphological and chemical information [116]. Beyond confocal microscopy, other depth-resolved Raman methods such as ultrafast time gated Raman spectroscopy [15,52,117] or spatial offset Raman spectroscopy (described above) [118,119] may have roles to play along these lines. As confocal Raman spectroscopy’s limited penetration depth may be as much as one order of magnitude lower than OCT, in the future it may be beneficial to combine OCT with SORS or the recently developed micro-SORS [120] to fully integrate these methods. 

## 5. Raman Plus Phase Microscopy

One of the key advantages of Raman scattering measurements, as discussed elsewhere, is that they are label-free. As such, they are very well suited to longitudinal measurements of dynamic processes. Another label-free modality is phase imaging. Phase imaging, pioneered by Frits Zernike (and earning him a Nobel Prize in 1953) [121,122], is a common technique in the life sciences to visualize optically transparent, thin specimens such as cells and tissue slices. As the light field propagates through such a sample, diffraction, scattering, and other processes alter the phase of that field. By interfering this altered field with light that has not passed through the sample, these phase alterations can be converted into intensity changes. However, like elastic scattering, phase contrast primarily reports on object structure, and carries little information about the chemical content. Thus, by combining phase microscopy and Raman together, label-free monitoring of dynamic cell process can be readily characterized.

The Li laboratory has long been exploring this avenue, combining Raman spectroscopy with phase contrast (and even fluorescence) microscopies to monitor dynamic changes in bacterial spores [123,124]. The water content in the core of dormant spores is very low and thus has lower refractive index compare to the core of the germinated spores. Then the alteration in refractive index during germination can be probed using phase-contrast microscopy [125]. However, phase-contrast microscopy alone cannot provide molecular changes during germination. By staining the nucleus with SYTO16, changes in the permeability of the cell wall during germination can be tracked as the dye enters the spore during germination leading to a time-dependent, strong fluorescence signal of the core versus time. Raman spectroscopy, meanwhile, is used to characterize and quantify pyridine-2,6-dicarboxylic acid (dipicolinic acid (DPA)) and its chelated divalent metal ions (predominantly Ca^2+^ (CaDPA)) in the core of a single spore. CaDPA is released from the spore and replaced with water during germination. This multi-parametric method allows them to simultaneously measure the loss of spore refractivity, changes of spore specific molecules and uptake of the nucleic acid stain in the process of germination. Using this system they have quantitatively analyzed the germination dynamics of *B. subtilis* and *C. difficile*, exploring the impacts of heat shock in germination [126] as well as exploring the changes in hydration within the bacterial core [127]. Using the combined phase and Raman information allowed them to demonstrate that the long germination times of so-called super-dormant species of *B. subtilis* is due to low concentrations of nutrient-binding receptors on the bacterial surface [128]. In another study, they measured the kinetics and levels of berberine accumulation by individual dormant and germinated spores [129]. Raman and phase measurements demonstrated that the level of the beberine accumulation reached a maximum after completion of CaDPA release, while fluorescence and phase imaging showed that berberine accumulated primarily in core of the germinated spores. 

In another study, Pavillon et al. demonstrated a multimodal system combining quantitative phase microscopy (QPM) and Raman microscopy to measure two independent properties of dynamic sample morphology and biomolecular information simultaneously [130]. QPM is a variant of traditional phase microscopy where the phase is quantitatively recovered, allowing more detailed study of sample morphology. Unlike Raman imaging, QPM can be done at video rate, and provides unique structural information that can be utilized alongside the chemical content provided by Raman imaging. They demonstrate, for example, that by rapidly acquiring QPM images of a large field of view, regions of interest can be identified for rapid acquisition of mean Raman spectra [131]. By rapidly scanning the laser beam across the ROI, more statistically consistent spectra (with higher SNR) can be obtained than by either Raman imaging or Raman point spectroscopy. McReynolds et al. also recently demonstrated the power of combining Raman and QPM for discrimination of immune cell subsets [132]. Similar to the report by Pavillon et al., a principal advantage is the speed of QPM vs. Raman, allowing for rapid identification of small ROIs that can be probed more carefully by Raman spectroscopy.

## 6. Raman Plus Mass Spectrometry

Another combination attracting recent attention is Raman combined with Mass spectrometry. Mass spectrometry (MS) is the technique of choice for discriminating between complex yet structurally similar molecules such as proteins. However, Raman spectroscopy has the ability to unambiguously identify regioisomers that cannot be separated by MS. However, because Raman and MS are governed by widely differing physical principles, not to mention that MS is a destructive method, their combination is not straightforward. Typical implementations require sequential measurement of the two modalities, and often they are not guaranteed to probe the same molecules or same volume. Nevertheless, these combination have great potential for certain bioanalytical applications. To fully exploit Raman and MS for bioassays, several researchers have developed unique multi-functional substrates that enhance both Raman and MS at the same time. In this direction, Meher et al. demonstrated Raman spectroscopy and MS of several chemicals in a single setup with tissue paper as substrate. The tissue paper on one hand provided a stable support for SERS nanoparticle-assisted Raman measurements, and subsequently provided support for low-background electrospray generation to feed the sample into a MS system. This development enabled them to detect and distinguish positional isomers and monitor complex reaction process with high time resolution. [133]. Another recent Raman/MS substrate is presented in a fascinating paper by Alessandri et al. Using a special all dielectric nanoparticle substrate (SiO_2_/TiO_2_ core/shell, AKA T-rex, nanoparticles), the authors demonstrate that the T-rex nanoparticles both provide plasmon-free SERS enhancement, while at the same time provide a background-free promotion of ionization via so-called Surface Laser Assisted Desorption/Ionization (SALDI). The multimodal SERS/SALDI platform (dubbed “RaMassay”) was used to identify two structurally related compounds difficult to discriminate by Raman, along with two regioisomers difficult to distinguish by MS [134]. These platforms have significant potential use in identification of illegal narcotics, small molecule drugs, reaction monitoring, and identification of explosive samples [135].

**Figure 4:**
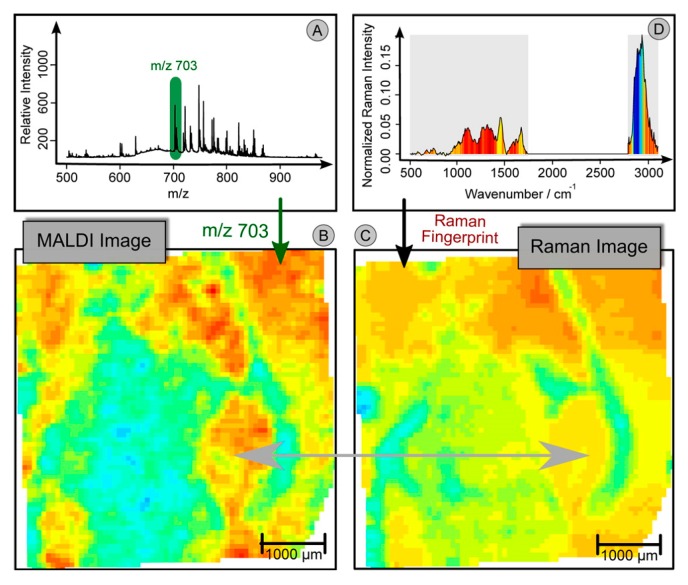
Workflow of Quantitative Correlation. (**A**) Mean MALDI spectrum of the (Raman) scanned region. (**B**) Integrating a peak region (*m*/*z* 703) leads to a false-color image, which can be interpreted as a chemical map. (**C**) By multivariate calibration methods, a model can be constructed, which translates a MALDI peak into a complex Raman signature. Here we used a PLS-model to construct a model for the MALDI marker of gray matter (*m*/*z* 703). The Raman signature can then be applied on sections without applying the MALDI imaging. This allows the use of information associated with the MALDI marker of gray matter (*m*/*z* 703) under noninvasive conditions or together with a contrary MALDI matrix. (**D**) The mean Raman spectrum of the region is given. The color inside the spectrum represents the translated Raman marker of gray matter. The Raman signature corresponds to the weights of the PLS-model, which was used to construct (C). Peaks which are red are mostly expressed in regions, where (B, C) show high values (red). On the other hand, the blue area in the CH-wavenumber region of (D) is related to the blue and green region of (B, C). Reprinted with permission from Bocklitz et al., [137]. Copyright 2013 American Chemical Society.

While the above research focuses on bulk analysis of sample material, higher resolution studies are also possible. For example, by combining Raman and fluorescence imaging with micro-droplet mass spectroscopy, researchers were able to acquire and correlate complementary information of biomolecules in individual algal cells undergoing a complex encystment process [136]. Fluorescence monitoring of chlorophyll tracked release of the chemical into cytosol for biotransformation to astaxanthin, whose accumulation was monitored by Raman spectroscopy. In addition, the authors detected 13 metabolites using MS, including ATP and ADP. Using these three pieces of information, they are able to correlate the accumulation of astaxanthin to a reduction in the ATP/ADP ratio, as expected due to a loss of chloroplast function in the ATP cycle. Furthermore, in an excellent study published recently by Bocklitz et al., high resolution MS mapping was combined with high resolution Raman mapping of tissue [137]. The MS and Raman maps were co-registered for enhanced multimodal visualization of chemical structures within tissue. Furthermore, the multiple modalities were leveraged in an intriguing way, dubbed “Quantitative Correlation”, as shown in Figure 4. Contrast obtained with mass spectroscopy and that obtained with Raman were quite different, due to their differing sensitivities to various compounds within the tissue. However, by using the spatial distribution of peaks in the mass spectra, Raman signatures could be obtained corresponding to those variations, enabling deeper understanding of the origin of the chemical signatures, and even predicting MS maps of images without needing to perform the MS experiment (or where contrary MALDI matrices are applied). This deep leveraging of multiple modalities demonstrates the full power of combining complementary methods, despite the substantial experimental heroics required to sequentially map and co-register MS and Raman signals of a microscopic region.

## 7. Perspective and Future Directions

As demonstrated in the preceding sections, Raman spectroscopy has an important role to play in biomedical research as well as clinical practice. The advent of CCD-based spectroscopy in the 1990s led to a swift uptick in Raman-based research, resulting in widespread enthusiasm about Raman spectroscopy’s promise for label free biosensing and as a noninvasive optical biopsy tool. Despite early enthusiasm, however, and mirroring the trajectories of many other biomedical technologies, 20 years later Raman has yet to transition into a routine tool in medical or biological contexts. This may be in part due to the institutionally slow adoption of new technologies by conservative medical professionals, but it is surely also due to some limitations in Raman spectroscopy (at least as it is currently performed). Thus, innovative combinations of Raman scattering with other modalities can help to expand the application space that can be successfully addressed by Raman technology.

A table providing a concise overview of the literature discussed here is presented in Table 1. Some of the particular highlights in the recent literature follow: the combination of Raman and fluorescence imaging by Kong and co-authors was able to speed up the analytical throughput of Raman-based margin-detection in frozen sections by several orders of magnitude, with no loss in analytical throughput. The addition of an elastic scattering channel to a Raman microspectroscopy system by Smith and Berger yielded sub-diffraction information about cellular morphology essentially “for free” by using elastically scattered light already generated by the Raman excitation laser. Combining Raman with OCT by Maher and co-authors allowed for quantitative determination of drug penetration in multi-layered tissue. These and other examples detailed above demonstrate the power of “Raman plus X”. Nevertheless, depending on the technologies being combined, certain caveats must be admitted.

Raman spectroscopy is most commonly performed in biomedical samples using near infrared wavelengths which may differ significantly from those employed by other optical technologies. Thus, the resolutions of the different systems have the potential to be quite different. Furthermore, when combining Raman with, say, OCT or even non-optical modalities, the probing depth or probing areas of the measurements may also be substantially different. A diffuse Raman measurement will probe a significantly larger volume than an OCT scan, which must be kept in mind when considering the correlation between these two distinct sources of information. 

Caveats aside, it is clear that Raman in combination with other modalities brings a whole greater than the sum of their parts. In the future we expect to see a continuation of the trend of Raman spectroscopy serving in concert with other methods rather than on its own, and that these combined methods will be what ultimately transforms Raman spectroscopy and imaging into a commonplace biological and clinical tool. Aside from continued translational work on systems already published, new methods are expected that may be extensions of prior work, such as joining Raman spectroscopy with other elastic scattering-based sizing methods (such as dynamic light scattering), or in as-yet untested combinations, such as Raman spectroscopy joined with partial wave spectroscopy and/or other emerging optical and non-optical methods in the life sciences.

## Figures and Tables

**Table 1:** Overview of multimodal Raman technologies.

Raman + X	Additional Information Provided	Biomedical Applications
Fluorescence	Chemical information of specific proteins or molecular targets	Cancer detection [23], arterial plaque evaluation [26], cancer margin assessment [28]
Elastic Scattering	Additional signal strength, layer sensitivity, reduction of fluorescence	Pharmaceutical quality control [57,58], bone quality [66,70,75], cancer diagnosis [81,83]
	Morphological Information	Cellular organization [93,94], cellular dynamics [96]
OCT	Tissue morphology, correlate depth-resolved Raman with tissue structure	Drug penetration [115,116], correlation of structure and function [114]
Phase imaging	Refractive index information, cellular morphology	Spore germination [123,127,128], cell identification and discrimination [132]
Mass spectrometry	Additional chemical detail	Illicit drug forensics [134], cellular mapping and analysis [136,137]
